# Low Dose Combined Treatment with Ultraviolet-C and Withaferin a Enhances Selective Killing of Oral Cancer Cells

**DOI:** 10.3390/antiox9111120

**Published:** 2020-11-13

**Authors:** Sheng-Yao Peng, Yen-Yun Wang, Ting-Hsun Lan, Li-Ching Lin, Shyng-Shiou F. Yuan, Jen-Yang Tang, Hsueh-Wei Chang

**Affiliations:** 1 Department of Biomedical Science and Environmental Biology, PhD Program in Life Science, College of Life Science, Kaohsiung Medical University, Kaohsiung 80708, Taiwan; u109851101@kmu.edu.tw; 2 School of Dentistry, College of Dental Medicine, Kaohsiung Medical University, Kaohsiung 80708, Taiwan; wyy@kmu.edu.tw (Y.-Y.W.); tinghsun.lan@gmail.com (T.-H.L.); 3 Center for Cancer Research, Kaohsiung Medical University, Kaohsiung 80708, Taiwan; yuanssf@kmu.edu.tw; 4 Cancer Center, Kaohsiung Medical University Hospital, Kaohsiung 80708, Taiwan; 5 Division of Prosthodontics, Department of Dentistry, Kaohsiung Medical University Hospital, Kaohsiung Medical University, Kaohsiung 80708, Taiwan; 6 Department of Radiation Oncology, Chi-Mei Foundation Medical Center, Tainan 71004, Taiwan; 8508a6@mail.chimei.org.tw; 7 School of Medicine, Taipei Medical University, Taipei 11031, Taiwan; 8 Chung Hwa University Medical Technology, Tainan 71703, Taiwan; 9 Translational Research Center, Kaohsiung Medical University Hospital, Kaohsiung 80708, Taiwan; 10 Department of Radiation Oncology, Faculty of Medicine, College of Medicine, Kaohsiung Medical University, Kaohsiung 80708, Taiwan; 11 Department of Radiation Oncology, Kaohsiung Medical University Hospital, Kaohsiung 80708, Taiwan; 12 Institute of Medical Science and Technology, National Sun Yat-sen University, Kaohsiung 80424, Taiwan

**Keywords:** ultraviolet-C (UVC), withanolide, combined treatment, oral cancer, DNA damage

## Abstract

Withaferin A (WFA), a *Withania somnifera*-derived triterpenoid, is an anticancer natural product. The anticancer effect of nonionizing radiation such as ultraviolet-C (UVC) as well as the combined treatment of UVC and WFA is rarely investigated. Low dose UVC and/or WFA treatments (12 J/m^2^ and/or 1 μM) were chosen to evaluate antioral cancer cell line effects by examining cytotoxicity, cell cycle disruption, apoptosis induction, and DNA damage. For two cancer cell lines (Ca9-22 and HSC-3), single treatment (UVC or WFA) showed about 80% viability, while a combined treatment of UVC/WFA showed about 40% viability. In contrast, there was noncytotoxicity to normal oral cell lines (HGF-1). Compared to single treatment and control, low dose UVC/WFA shows high inductions of apoptosis in terms of flow cytometric detections for subG1, annexin V, pancaspase changes as well as Western blotting for detecting cleaved poly (ADP-ribose) polymerase (c-PARP) and caspase 3 (c-Cas 3) and luciferase assay for detecting Cas 3/7 activity. Low dose UVC/WFA also showed high inductions of oxidative stress and DNA damage in terms of flow cytometric detections of reactive oxygen species (ROS), mitochondrial superoxide (MitoSOX) generation, and membrane potential (MitoMP) destruction, γH2AX and 8-oxo-2’deoxyguanosine (8-oxodG) types of DNA damages. For comparison, low dose UVC/WFA show rare inductions of annexin V, Cas 3/7 activity, ROS, MitoSOX, and MitoMP changes to normal oral HGF-1 cells. Therefore, low dose UVC/WFA provides a novel selectively killing mechanism to oral cancer cells, suggesting that WFA is a UVC sensitizer to inhibit the proliferation of oral cancer cells.

## 1. Introduction

Radiotherapy and chemotherapy are commonly used for curing oral cancer [1]. However, they also raise the problems of radio- and chemo-resistance [2]. Recently, a novel strategy was developed to reduce chemoresistance by using low dose treatment. For example, in the clinical drug doxorubicin, its low dose treatment improved survival by suppressing the patient-derived chemoresistant leukemia stem cells in an animal model study [3]. Accordingly, low dose drug treatments have a potential for reducing chemoresistance in oral cancer therapy.

Similarly, high dose radiation is commonly associated with radioresistance to cancer such as prostate cancer cells [4]. Low dose radiation treatments have a potential for reducing radioresistance in cancer therapy. In addition to dose adjustments of radiation, a combined treatment provides an alternative strategy to overcome radioresistance in cancer therapy. For example, DNA-dependent protein kinase (DNA-PK) inhibitors KU57788 and IC87361 [5] and multikinase inhibitors sorafenib and sunitinib [6] displayed radiosensitization to head and neck cancer cells.

In addition to X-ray radiotherapy for oral cancer, several types of nonionizing radiation were developed for dental curing, including light-emitting diodes (LED), quartz-tungsten-halogen (QTH), argon lasers, and plasma arc curing (PAC) [7]. Except for dental curing, another nonionizing radiation such as ultraviolet-C (UVC) is commonly used for its germicidal properties on dental instruments [8]. Based on Google scholar and PubMed searching (retrieval on 7 September 2020), the hits for “X-ray resistance” studies were higher than “UVC resistance”. Therefore, the combined treatment of UVC and drugs may have potential to improve the anticancer therapy. Moreover, UVC is also reported to have an anticancer effect against pancreatic [9] and colon [10] cancer cells. However, high cytotoxic doses were chosen in the above studies and may have some side effects. Accordingly, low doses of UVC may reduce the side effects to normal tissues.

Withaferin A (WFA), a triterpenoid isolated from *Withania somnifera*, shows antioxidant properties [11,12]. In addition to antioxidant effect, WFA also exhibits anticancer effects by showing cytotoxicity and inducing apoptosis to oral [13,14] and other types of cancer cells [15,16,17,18]. However, the observed cytotoxic effects of WFA in anticancer studies were based on high doses. Recently, combined treatments of WFA with other drugs have been reported. For example, the combined treatment of WFA and oxaliplatin showed synergistic antitumor activity in pancreatic cancer cells [16]. WFA was also combined with caffeic acid phenethyl ester (CAPE) for selective toxicity for ovarian and cervical cancer cells [19]. However, the combined treatment of WFA with UVC irradiation is rarely investigated.

We hypothesized that low dose WFA had an UVC radiosensitizing effect to oral cancer cells. To address this hypothesis, a low dose combined treatment (UVC/WFA) and individual treatments of oral cancer cells were compared for their changes of cytotoxicity, cell cycle, apoptosis, oxidative stress, and DNA damage.

## 2. Materials and Methods

### 2.1. Cell Cultures and Chemical

Two human oral cancer cell lines (Ca9-22 and HSC-3) and normal oral cell lines (HGF-1) were commercially obtained from the Japanese Collection of Research Bioresources (JCRB) Cell Bank (National Institute of Biomedical Innovation, Osaka, Japan) and the American Type Culture Collection (ATCC; Manassas, VA, USA). Ca9-22 cells were derived from gingival squamous carcinoma. HSC-3 cells were derived from tongue squamous carcinoma with high metastatic potential. HGF-1 cells were derived from gingival fibroblasts. The relative proliferation rate for Ca9-22, HSC-3, and HGF-1 cells was 1.9:1.6:1.0, respectively [20]. Their routinely cultured methods were described as indicated in [21]. WFA was obtained from Sigma-Aldrich (St. Louis, MO, USA). Cisplatin (Selleckchem; Houston, TX, USA) and H_2_O_2_ (Sigma-Aldrich; St. Louis, MO, USA) were used as a positive control treatment.

### 2.2. Cytotoxicity, ATP Content Determination, and Cell Morphology

Cytotoxicity was evaluated by tetrazolium-based 3-(4,5-dimethylthiazol-2-yl)-5-(3-carboxymethoxyphenyl)-2-(4-sulfophenyl)-2H-tetrazolium (MTS) kit (Promega Corporation, Madison, WI, USA) as described previously [22]. The cell viability in terms of cellular ATP content was evaluated by an ATP-lite assay kit (PerkinElmer Life Sciences, Boston, MA, USA) [23]. The drug interaction for the UVC/WFA combined treatment was analyzed as previously described [24]. In brief, the formula for determining the synergy (α), i.e., additive, synergistic, or antagonistic for α = 1, >1, and <1, respectively, was listed as follows: α = survival fraction (SF) for UVC alone treatment × SF for WFA alone treatment/SF for UVC/WFA combined treatment. Cells were photographed at 100× magnification for morphology analysis.

### 2.3. UVC and/or WFA Treatments

After removing medium, cells were treated with a germicidal UVC lamp (254 nm) in a laminar flow chamber at a rate of 2 J/m^2^/sec [25] for 6 sec to archive the designed dose 12 J/m^2^. Subsequently, medium containing WFA was added for post-treatment. The control contained DMSO only without UVC and WFA treatments. All experiments including DMSO only, UVC and/or WFA treatments had the same concentration of 0.01% DMSO.

### 2.4. Cell Cycle

DNA was stained with 7-Aminoactinmycin D (7AAD) (Biotium, Inc., Hayward, CA, USA) (1 μg/mL, 37 °C, 30 min) for a cell cycle assay [26]. Flow cytometer (Guava easyCyte; Luminex, TX, USA) and FlowJo software (Becton-Dickinson; Franklin Lakes, NJ, USA) were applied. Both subG1 and >4 N populations were individually counted. G1, S, and G2/M populations were totally adjusted to 100%.

### 2.5. Apoptosis

A flow cytometer (Guava easyCyte) applying FlowJo software (Becton-Dickinson) was used to measure apoptosis in terms of annexin V (Strong Biotect Corporation, Taipei, Taiwan)/7AAD [27] and a pancaspase activity assay kit (Abcam, Cambridge, UK) [23] assays. Apoptosis was also detected by Western blotting applying primary antibodies for the cleaved forms of poly (ADP-ribose) polymerase (c-PARP) and caspase 3 (c-Cas 3) (Cell Signalling Technology Inc., Danvers, MA, USA) (diluted 1:1000) and internal control mAb-β actin (Sigma-Aldrich, St. Louis, MO, USA) as described previously [14]. Cas 3/7 activity was determined by a Caspase-Glo^®^ 3/7 Assay (Promega; Madison, WI, USA) based on luminescent detection as described previously [28]. 

### 2.6. Reactive Oxygen Species (ROS)

Reactive species-detecting probe 2’,7’-dichlorodihydrofluorescein diacetate (DCFH-DA) (10 μM, 37 °C, 30 min) was interacting with ROS to generate fluorescence, which was determined by a flow cytometer (Guava easyCyte) applying FlowJo software (Becton-Dickinson) [29].

### 2.7. Mitochondrial Superoxide (MitoSOX)

Reactive species-detecting probe MitoSOX Red (Molecular Probes, Invitrogen, Eugene, OR, USA) (50 nM, 37 °C, 30 min) was interacting with MitoSOX to generate fluorescence, which was determined by a flow cytometer (Guava easyCyte) applying FlowJo software (Becton-Dickinson) [30].

### 2.8. Mitochondrial Membrane Potential (MitoMP)

MitoMP-sensitive probe DiOC_2_(3) (Invitrogen, Eugene, OR, USA) (5 nM, 37 °C, 30 min) was used to determine MitoMP by a flow cytometer (Guava easyCyte) applying FlowJo software (Becton-Dickinson) [31]. 

### 2.9. γH2AX

After cell harvesting and fixation, γH2AX primary antibody (Santa Cruz Biotechnology; Santa Cruz, CA, USA) (1:500 dilution), secondary antibody conjugated Alexa Fluor^®^488 (Cell Signaling Technology) (1:10000 dilution), and 7AAD (5 μg/mL) were chosen for flow cytometry reaction [32] and analyzed by a flow cytometer (Guava easyCyte) applying FlowJo software (Becton-Dickinson). By Western blotting, γH2AX was also probed using primary antibodies for γH2AX (Santa Cruz Biotechnology; Santa Cruz, CA, USA) (diluted 1:1000).

### 2.10. 8-Oxo-2’-Deoxyguanosine (8-OxodG)

After cell harvesting and fixation, fluorescein isothiocyanate (FITC) conjugated 8-oxodG antibody (1:10,000 dilution) (Santa Cruz Biotechnology) were chosen for flow cytometry reaction at 4 °C for 1 h and analyzed by a flow cytometer (Guava easyCyte) applying FlowJo software (Becton-Dickinson).

### 2.11. Statistics

The significance for multiple comparisons were tested by One-way ANOVA with the Tukey Honestly Significant Difference (HSD) post hoc test using JMP12 software (SAS Institute, Cary, NC, USA). Groups showing no overlapping letters indicate significant differences.

## 3. Results

### 3.1. WFA Shows UVC Sensitizing Effects on Cytotoxicity of Oral Cancer Cells

**Figure 1:**
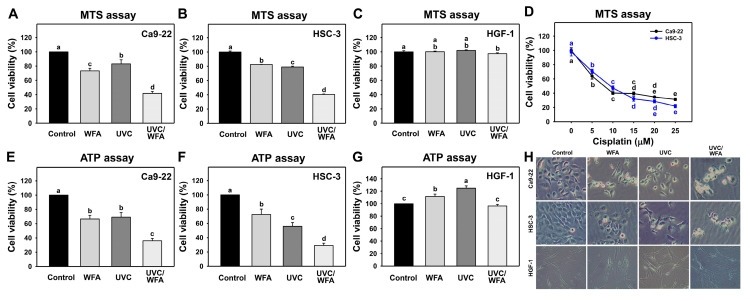
MTS and ATP assays for Withaferin A (WFA) and/or UV treatments. Human oral cancer Ca9-22 and HSC-3 cells and normal oral HGF-1 cells were treated with control (0.01% DMSO), WFA (1 µM), ultraviolet-C (UVC) (12 J/m^2^), and a combined treatment (UVC/WFA) or cisplatin for 24 h. (**A–C**) MTS assay for WFA treatment. (**D**) MTS assay for a positive cisplatin control. (**E**–**G**) ATP assay for WFA treatment. Groups showing no overlapping letters (a–e) indicate significant differences (*p* < 0.05~0.0001). Data are the mean ± SD (*n* = 3 independent experiments, each experiment was performed with three replications). (**H**) Cell morphology. Cell images were photographed at 100× magnification.

Based on the 24-h MTS assay, low cytotoxic UVC and WFA (12 J/m^2^ and 1 μM) around 80% viability were used to evaluate the cytotoxic effect of a combined treatment (UVC/WFA) of oral cancer Ca9-22 and HSC-3 cells and normal oral HGF-1 cells (Figure 1). As shown in Figure 1A, UVC/WFA-treated Ca9-22 cells show a lower viability of 42.2% than UVC or WFA alone (73.7% or 83.4%) in a 24-h MTS assay. As shown in Figure 1B, UVC/WFA-treated HSC-3 cells show lower viability for 40.6% than UVC or WFA alone (82.7% or 79.4%) in a 24-h MTS assay. In contrast, UVC/WFA shows no cytotoxicity towards normal oral HGF-1 cells (Figure 1C).

The interaction effects of UVC and WFA for Figure 1A,B (MTS assay (α values were 1.46 ± 0.14 and 1.11 ± 0.03 for Ca9-22 and HSC-3 cells, respectively)) and for Figure 1E,F (ATP assay (α values were 1.28 ± 0.09 and 1.39 ± 0.15 for Ca9-22 and HSC-3 cells, respectively)) show synergistic behavior. For comparison, cisplatin shows cytotoxicity towards oral cancer cells with less drug sensitivity than UVC/WFA (Figure 1D). Similarly, UVC/WFA-treated oral cancer Ca9-22 and HSC-3 cells show lower viability than UVC or WFA alone in a 24-h ATP assay (Figure 1E,F). Similarly to the MTS assay, UVC/WFA shows no cytotoxicity to normal oral HGF-1 cells in terms of ATP assay (Figure 1G). Furthermore, the UVC and/or WFA treatments show the apoptosis-like morphology such as cell shrinkages for oral cancer cells but not for oral normal cells (Figure 1H).

### 3.2. WFA Shows UVC Sensitizing Effect on Cell Cycle Disturbance of Oral Cancer Cells

**Figure 2:**
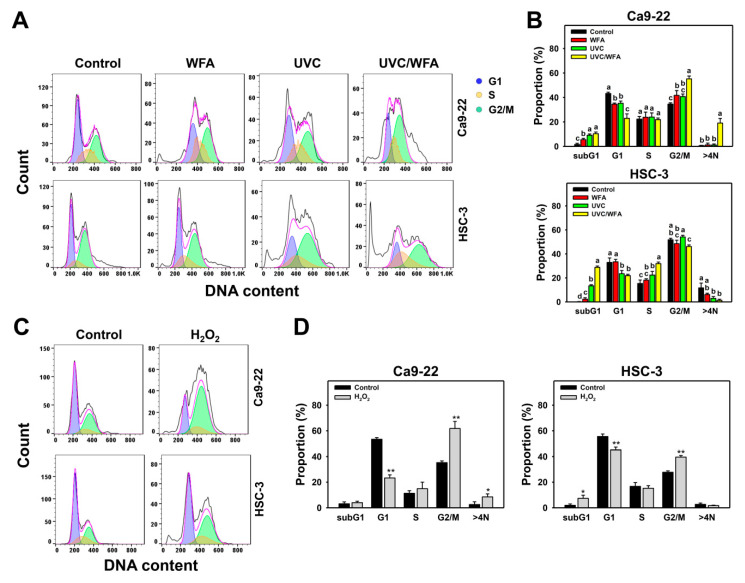
Cell cycle assay for WFA and/or UV treatments. Human oral cancer Ca9-22 and HSC-3 cells were treated with control (0.01% DMSO), WFA (1 µM), UVC (12 J/m^2^), and a combined treatment (UVC/WFA) for 24 h. (**A**,**B**) Typical cell cycle patterns and statistics. Groups showing no overlapping letters (a–d) indicate significant differences (*p* < 0.05~0.0001). Data are the mean ± SD (*n* = 3 independent experiments, each experiment collected with 5000 gated cell counts). (**C**,**D**) Cell cycle patterns for a positive control of G2/M arrest. Cells were treated with H_2_O_2_ for 0 and 200 µM for 24 h. *, ** *p* < 0.05~0.0001. Data are the mean ± SD (*n* = 3 independent experiments, each experiment collected with 5000 gated cell counts).

Figure 2A shows the cell cycle assays of oral cancer Ca9-22 and HSC-3 cells following 24-h treatments with control, WFA (1 µM), UVC (12 J/m^2^), or UVC/WFA. For Ca9-22 cells (Figure 2B), 24-h UVC/WFA treatment induces higher sub-G1, G2/M, and > 4N populations (%) than UVC, WFA, and the control. For HSC-3 cells, 24-h UVC/WFA treatment induces higher sub-G1 and S populations (%) than UVC, WFA, and the control. In contrast, G1 populations (%) of UVC/WFA for oral cancer Ca9-22 and HSC-3 cells are lower than UVC, WFA, and the control. For comparison, H_2_O_2_ shows G2/M arrest in oral cancer cells as a positive control (Figure 2C,D).

### 3.3. WFA Shows UVC Sensitizing Effect on Annexin V Expression and Caspase Activation of Oral Cancer Cells

**Figure 3:**
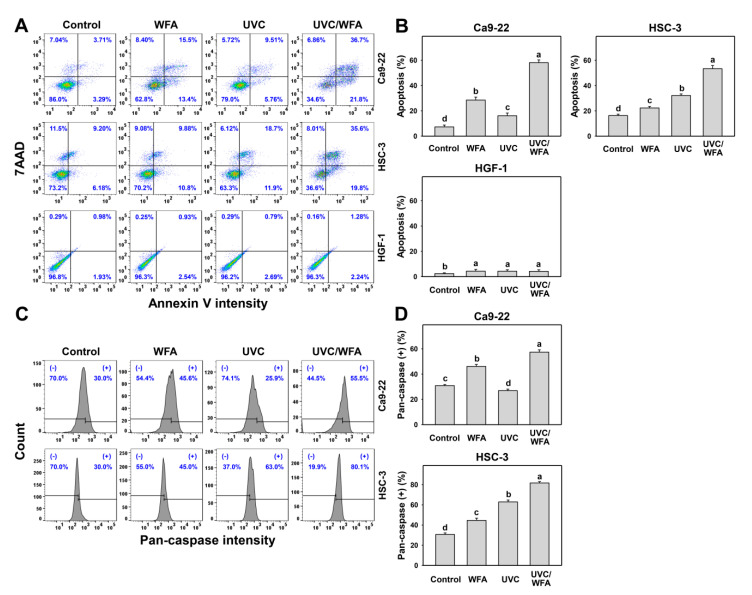
Annexin V and pancaspase assays of WFA and/or UV treatments. Human oral cancer Ca9-22 and HSC-3 cells and normal oral HGF-1 cells were treated with control (0.01% DMSO), WFA (1 µM), UVC (12 J/m^2^), and a combined treatment (UVC/WFA) for 24 h. (**A**,**B**) Typical annexin V/7AAD patterns and statistics. Apoptosis (%) is the percentage of annexin V-positive population. (**C**,**D**) Typical pancaspase pattern and statistics. (+) is the percentage for pancaspase-positive populations. Groups showing no overlapping letters (a–d) indicate significant differences (*p* < 0.05~0.0001). Data are the mean ± SD (*n* = 3 independent experiments, each experiment collected with 5000 gated cell counts).

The apoptosis-like status for increasing subG1 (Figure 2) was further examined by other apoptosis analyses as follows. According to an annexin V/7AAD assay (Figure 3A), 24-h UVC/WFA treatment induces higher annexin V (+) (%) populations in oral cancer Ca9-22 and HSC-3 cells than UVC, WFA, and control treatments (Figure 3B). In contrast, UVC and/or WFA treatments to normal oral HGF-1 cells show little annexin V (+) (%) populations.

**Figure 4:**
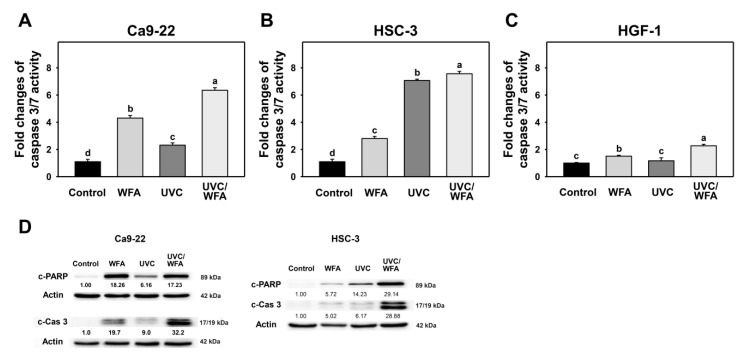
Caspase and PARP activations of WFA and/or UV treatments. Human oral cancer Ca9-22 and HSC-3 cells and normal oral HGF-1 cells were treated with control (0.01% DMSO), WFA (1 µM), UVC (12 J/m^2^), and a combined treatment (UVC/WFA) for 24 h. (**A**–**C**) Caspase 3/7 activity for Ca9-22, HSC-3, and HGF-1 cells. Groups showing no overlapping letters (a–d) indicate significant differences (*p* < 0.05~0.0001). Data are the mean ± SD (*n* = 3 independent experiments, each experiment was performed with three replications). (**D**) Western blotting for apoptotic protein c-PARP and c-Cas 3 expressions of oral cancer (Ca9-22 and HSC-3) cells.

According to a pancaspase assay (Figure 3C), UVC/WFA induces higher pancaspase (+) (%) populations in oral cancer Ca9-22 and HSC-3 cells than UVC, WFA, and the control (Figure 3D). Based on Cas 3/7 activity, UVC/WFA also induces higher Cas 3/7 activity in oral cancer and normal oral cells than UVC, WFA, and the control (Figure 4A–C). It is noted that UVC/WFA shows higher Cas 3/7 activity in oral cancer Ca9-22 and HSC-3 cells than normal oral HGF-1 cells.

According to Western blotting (Figure 4D), UVC/WFA induces higher expressions for the apoptotic protein such as the cleaved form of c-PARP and c-Cas 3 in oral cancer Ca9-22 and HSC-3 cells than UVC, WFA, and control.

### 3.4. WFA Shows UVC Sensitizing Effect on ROS Generation of Oral Cancer Cells

**Figure 5:**
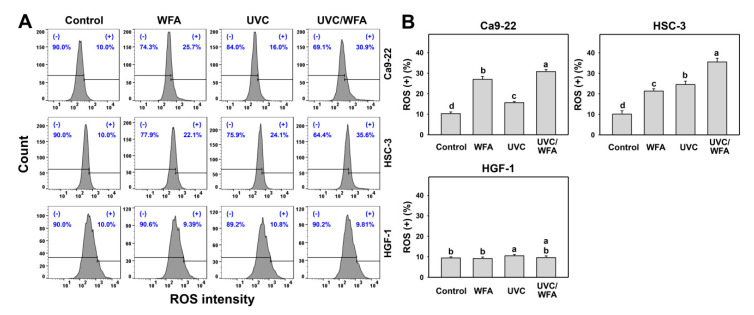
Reactive oxygen species (ROS) assays of WFA and/or UV treatments. Human oral cancer Ca9-22 and HSC-3 and normal oral HGF-1 cells were treated with control (0.01% DMSO), WFA (1 µM), UVC (12 J/m^2^), and a combined treatment (UVC/WFA) for 12 h. (**A**,**B**) Typical ROS patterns and statistics. (+) is the percentage for ROS-positive populations. Groups showing no overlapping letters (a–d) indicate significant differences (*p* < 0.05~0.0001). Data are the mean ± SD (*n* = 3 independent experiments, each experiment collected with 5000 gated cell counts).

Many factors such as oxidative stresses may induce apoptosis [33]. Because ROS generation following UVC [34], WFA [14], or UVC/natural product [35] treatments are fast, the ROS detection time of WFA/UVC was chosen as 12 h. Using DCFH-DA staining, the ROS generation was detected as oxidative stress by flow cytometry. According to an ROS assay for oral cancer Ca9-22 and HSC-3 cells (top and middle, Figure 5A), 12-h UVC/WFA treatment induces higher ROS (+) (%) populations than UVC, WFA, and control treatments (Figure 5B). In contrast, a ROS assay for normal oral HGF-1 cells (bottom, Figure 5A) shows no significant difference between UVC/WFA and single treatment, indicating that ROS is unable to be induced in WFA and/or UVC-treated normal oral HGF-1 cells.

### 3.5. WFA Shows UVC Sensitizing Effect on MitoSOX Generation of Oral Cancer Cells

**Figure 6:**
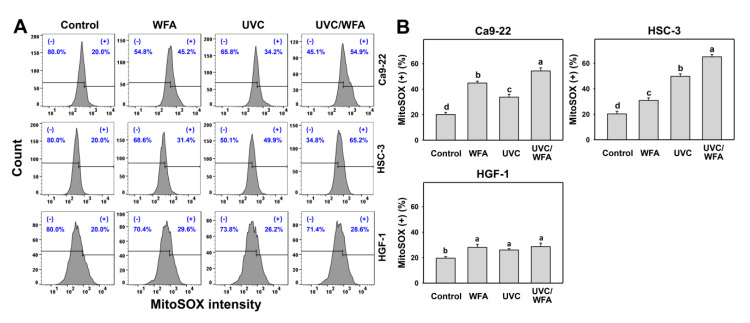
Mitochondrial superoxide (MitoSOX) assays of WFA and/or UV treatments. Human oral cancer Ca9-22 and HSC-3 cells and normal oral HGF-1 cells were treated with control (0.01% DMSO), WFA (1 µM), UVC (12 J/m^2^), and a combined treatment (UVC/WFA) for 24 h. (**A**,**B**) Typical MitoSOX patterns and statistics. (+) is the percentage for MitoSOX-positive populations. Groups showing no overlapping letters (a–d) indicate significant differences (*p* < 0.01~0.0001). Data are the mean ± SD (*n* = 3 independent experiments, each experiment collected with 5000 gated cell counts).

Using MitoSOX red staining, the MitoSOX generation was detected as oxidative stress by flow cytometry. According to a MitoSOX assay for oral cancer Ca9-22 and HSC-3 cells (top and medium, Figure 6A), 24-h UVC/WFA treatment induces higher MitoSOX (+) (%) populations than UVC, WFA, and control treatments (Figure 6B). In contrast, a MitoSOX assay for normal oral HGF-1 cells (bottom, Figure 6A) show no significant difference between UVC/WFA and single treatment. Moreover, WFA/UVC induces higher MitoSOX in oral cancer Ca9-22 and HSC-3 cells than in normal oral HGF-1 cells.

### 3.6. WFA Shows UVC Sensitizing Effect on MitoMP Destruction of Oral Cancer Cells

**Figure 7:**
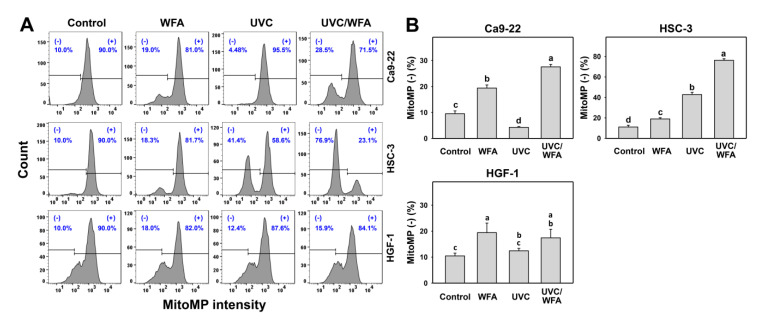
Membrane potential (MitoMP) assays of WFA and/or UV treatments. Human oral cancer Ca9-22 and HSC-3 cells and normal oral HGF-1 cells were treated with control (0.01% DMSO), WFA (1 µM), UVC (12 J/m^2^), and a combined treatment (UVC/WFA) for 24 h. (**A**,**B**) Typical MitoMP patterns and statistics. (-) is the percentage for MitoMP-negative populations. Groups showing no overlapping letters (a–d) indicate significant differences (*p* < 0.05~0.0001). Data are the mean ± SD (*n* = 3 independent experiments, each experiment collected with 5000 gated cell counts).

Using DiOC_2_(3) staining, the MitoMP depletion was detected as oxidative stress by flow cytometry. According to a MitoMP assay for oral cancer Ca9-22 and HSC-3 cells (top and medium, Figure 7A), 24-h UVC/WFA treatment induces higher MitoMP (-) (%) populations than UVC, WFA, and control treatments (Figure 7B). In contrast, a MitoMP assay for normal oral HGF-1 cells (bottom, Figure 7A) shows no significant difference between UVC/WFA and single treatment. Moreover, WFA/UVC induces higher MitoMP (-) (%) populations in oral cancer Ca9-22 and HSC-3 cells than in normal oral HGF-1 cells. 

### 3.7. WFA Shows UVC Sensitizing Effect on γH2AX and 8-oxodG Expressions of Oral Cancer Cells

**Figure 8:**
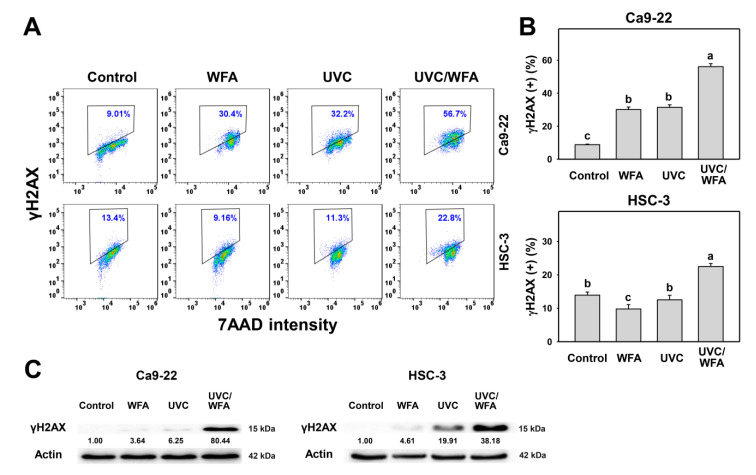
γH2AX assays of WFA and/or UV treatments. Human oral cancer Ca9-22 and HSC-3 cells were treated with control (0.01% DMSO), WFA (1 µM), UVC (12 J/m^2^), and a combined treatment (UVC/WFA) for 24 h. (**A**,**B**) Typical γH2AX patterns and statistics. Box indicates the percentage for γH2AX-positive populations. Groups showing no overlapping letters (a–c) indicate significant differences (*p* < 0.01~0.0001). Data are the mean ± SD (*n* = 3 independent experiments, each experiment collected with 5000 gated cell counts). (**C**) Western blotting for γH2AX expression.

**Figure 9:**
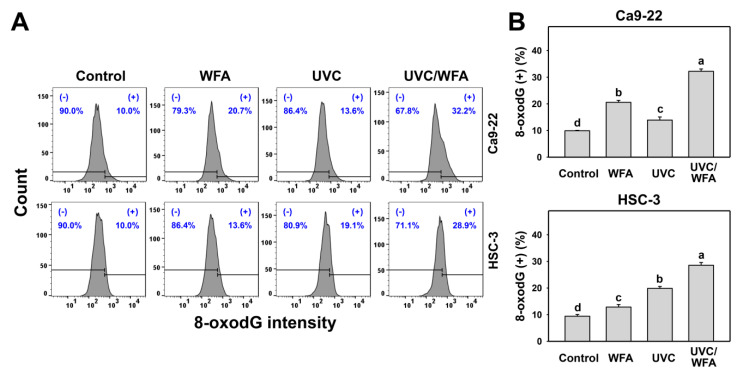
8-oxo-2’deoxyguanosine (8-oxodG) assays of WFA and/or UV treatments. Human oral cancer Ca9-22 and HSC-3 cells. Cells were treated with control (0.01% DMSO), WFA (1 µM), UVC (12 J/m^2^), and a combined treatment (UVC/WFA) for 24 h. (**A**,**B**) Typical 8-oxodG patterns and statistics. (+) is the percentage for 8-oxodG-positive populations. Groups showing no overlapping letters (a–d) indicate significant differences (*p* < 0.0001). Data are the mean ± SD (*n* = 3 independent experiments, each experiment collected with 5000 gated cell counts).

Since oxidative stress is prone to induce DNA damage [33], the changes of DNA damage following UVC and/or WFA treatment were detected by flow cytometry and Western blotting. According to a γH2AX assay (Figure 8A), 24-h UVC/WFA treatment induces higher γH2AX (+) (%) populations in oral cancer Ca9-22 and HSC-3 cells than UVC, WFA, and control treatments (Figure 8B). According to Western blotting (Figure 8C), UVC/WFA induces higher expressions for γH2AX in oral cancer Ca9-22 and HSC-3 cells than UVC, WFA, and the control. According to an 8-oxodG assay (Figure 9A), 24-h UVC/WFA treatment induces higher 8-oxodG (+) (%) populations in oral cancer Ca9-22 and HSC-3 cells than UVC, WFA, and the control (Figure 9B).

## 4. Discussion

WFA demonstrated X-ray radiosensitizing ability in many cancer cells [36,37,38] but its UVC sensitizing effect is rarely reported. UVC generation is more easy to handle with a user-friendly device than X-ray. Therefore, the present study focused on exploring the possible UVC sensitizing effect of WFA with cell line models. To avoid the potential resistance of high dose WFA and UVC, this study was evaluating low dose combined effects of UVC/WFA to oral cancer cells.

### 4.1. Combined Treatment UVC/WFA Effectively Kills Oral Cancer Cells without Side Effect to Normal Oral Cells

Low dose ionizing radiation and low dose drug treatment are novel strategies to reduce radioresistance and chemoresistance [3,4]. A combined treatment of radiation with drugs would also provide a strategy to overcome radioresistance and would improve cancer therapy [5,6].

Recently, the radiosensitizing studies using low dose clinical drugs and UVC were reported. For example, low dose cisplatin (10 μM) and UVC (10 J/m^2^) jointed to induce antiproliferation of colon cancer SW480 and DLD-1 cells [39]. Combined treatment with low dose methanolic extracts of *Cryptocarya concinna* (10 μg/mL; 80.4% viability) and UVC (14 J/m^2^; 83.2% viability) improve antiproliferation against oral cancer cells compared to single treatment [25].

WFA was previously shown to have no cytotoxicity towards normal oral HGF-1 cells at a low dose below 3 μM [14], while it showed cytotoxicity to oral cancer Ca9-22 and CAL 27 cells below 3 μM. Therefore, oral cancer therapy would benefit by the selective killing effect of WFA. Alternatively, in the present study this low dose treatment was combined with nonionizing radiation, i.e., UVC. Combining these strategies, we demonstrated that a low dose combined treatment of UVC/WFA dramatically decreased cell viability (~40%) of two oral cancer cell lines (Ca9-22 and HSC-3) without cytotoxic effects on normal oral cells in a 24-h MTS assay (Figure 1). 

Consistently, UVC/WFA shows substantially decreased ATP content in oral cancer cells but does not decrease in oral normal cells. Moreover, the half maximal inhibitory concentration (IC_50_) values of cisplatin to oral cancer Ca9-22 and HSC-3 cells in a 24-h MTS assay are 7.9 and 9.6 μM, respectively. At a low dose, UVC (12 J/m^2^) and WFA (1 µM) were used in the present study to demonstrate a higher antiproliferation ability to oral cancer Ca9-22 and HSC-3 cells (42.2% and 40.6% viability, respectively) than cisplatin alone. It is noted that the oral cancer Ca9-22 cell line is recently described for being problematic in terms of showing contamination with the cell line MSK922 (according to the Cellosaurus database [40]). It warrants detailed investigation by applying other oral cancer cell lines to support the UVC radiosensitizing effect of WFA in the future. Moreover, the HGF-1 cell line is derived from fibroblasts rather than from normal epithelial cells. The human oral keratinocytes would be a better control to show that the suppression of cell viability and induction of apoptosis after UVC/WFA treatment is cancer cell specific.

WFA shows antioxidant properties such as 1,1-diphenyl-2-picrylhydrazyl (DPPH) radical scavenging ability in vitro [12]. WFA also showed in vivo antioxidant properties with activation of superoxide dismutase (SOD), catalase (CAT), and glutathione peroxidase (GPX) in the frontal cortex of the rat brain [11]. Antioxidants are commonly reported to prevent oxidative stress-related damage [41]. In addition to WFA, the roots of *Withania somnifera* also contain several WFA analogues such as 1-oxo-5beta and 6beta-epoxy-witha-2-enolide, which prevent the UVB-induced skin carcinoma in rat [42,43]. In contrast, WFA inhibited proliferation of oral [14] and lung [17] cancer cells as well as leukemia cells [44] through oxidative stress induction. Similarly, our finding shows that WFA in combination with UVC can inhibit more cell proliferation than its single treatment. This controversy can be partly explained by the concept that antioxidants are bifunctional to regulate oxidative stress, i.e., low concentration inhibits oxidative stress but high concentration induces oxidative stress [32,45].

### 4.2. Oxidative Stresses Are Higher in a Combined UVC/WFA Than in Single Treatment

ROS modulating is one of the strategies for antiproliferation of cancer cells [33]. Both high dose UVC (200 J/m^2^) [46] and WFA [14,18] trigger ROS in cancer cells. For low dose treatment, UVC (14 J/m^2^ [25] and 12 J/m^2^ (Figure 1); ~80% viability) induces ROS generation of oral cancer Ca9-22 and HSC-3 cells. For low dose treatment, WFA (0.5 μM [47] for 100% viability and 1 μM (Figure 1) for ~80% viability) also triggers ROS generation of oral cancer Ca-22 and HSC-3 cells. At a low dose combined treatment, UVC/WFA triggers more ROS generation than single treatment. Similarly, UVC/WFA triggers more MitoSOX generation and MitoMP destruction than single treatment (Figure 5, Figure 6 and Figure 7). These results suggest that UVC/WFA cooperatively triggers oxidative stress in oral cancer cells compared to single treatment. Moreover, UVC/WFA triggers more ROS and MitoSOX generation as well as MitoMP destruction in oral cancer cells than in normal oral HGF-1 cells. Although some of these oxidative stress changes for UVC/WFA were only slightly higher than at single treatment, the combined differential oxidative stresses (ROS/MitoSOX generations and MitoMP depletion) may cooperatively contribute to the cancer cell specific effects of a UVC/WFA combined treatment.

### 4.3. DNA Damages and Apoptosis Are Higher in UVC/WFA than Single Treatment

Cellular oxidative stress enhances DNA damages [33]. High dose UVC causes γH2AX-detecting DNA double strand break damage [48] and 8-oxodG-detecting oxidative DNA damage [49]. High dose WFA causes γH2AX DNA damage [14]. In the present study, low dose UVC/WFA triggers more DNA damages (γH2AX and 8-oxodG) in oral cancer Ca9-22 and HSC-3 cells than single treatment (Figure 8 and Figure 9). These results suggest that UVC/WFA cooperatively trigger DNA damage in oral cancer cells compared to single treatment. In addition to DNA damage induction, γH2AX also functions for senescence induction [50]. Our present flow cytometry approach could not discriminate between these two effects. A promising alternative would be a detailed investigation by immunofluorescence microscopy analyzing DNA damage foci formation in the future.

High dose UVC [25,51] or WFA [14,18] causes apoptosis in cancer cells. Similarly, low dose UVC/WFA triggers more apoptosis than single treatment as evidenced by flow cytometry or luminescent detection indicating an increasing subG1 population, annexin V expression, pancaspase activation, and Cas 3/7 activation as well as by Western blotting indicating c-PARP and c-Cas 3 overexpression in oral cancer cells (Figure 2, Figure 3 and Figure 4). Moreover, UVC/WFA triggers more Cas 3/7 activation in oral cancer cells than in HGF-1 cells (Figure 4). Additionally, the loss of the mitochondrial membrane potential is often associated with early stages of apoptosis [52]. Similarly, a combination of low dose UVC/WFA triggers more mitochondrial membrane depolarization in oral cancer cells than single treatment (Figure 7), supporting also that low dose UVC/WFA triggers more apoptosis than single treatment.

Furthermore, the DNA repair system may be inhibited for drug radiosensitization. For example, withanolide D is helpful in radiosensitization of ovarian cancer SKOV3 cells by suppressing DNA repair such as non-homologous end joining (NHEJ). This warrants a detailed examination of the role of DNA repair in UVC/WFA synergistic effects to oral cancer cells in the future.

### 4.4. Cell Cycle Changes at UVC and/or WFA Treatment

UVC/WFA accumulated a larger subG1 population than single treatment in both oral cancer Ca9-22 and HSC-3 cells. However, UVC/WFA induced more G2/M arrest in Ca9-22 cells than upon single treatment but no G2/M arrest appeared in HSC-3 cells. Because Ca9-22 and HSC-3 cells are gingiva and tongue squamous cell carcinoma cell lines [53], it is possible that UVC/WFA-induced cell cycle G2/M arrest is tissue-dependent response.

## 5. Conclusions

The present study examined the combined effect of low dose UVC/WFA on regulating cell proliferation of oral cancer cells. Single treatment at low dose UVC or WFA showed low cytotoxicity to oral cancer Ca9-22 and HSC-3 cells. Combined treatment of low dose UVC/WFA highly induces oxidative stress and resulted in apoptosis and DNA damage of oral cancer cells. Moreover, low dose UVC/WFA showed highly cytotoxic, oxidative stress, and apoptosis induction in oral cancer cells without effects on normal oral cells. Therefore, low dose UVC/WFA shows a selective killing potential and effectively inhibits oral cancer cell proliferation with no cytotoxic side effects to normal oral cells. In the future, detailed investigations in vivo, such as those using mouse models, should be conducted to further validate the promising effects of a low dose UVC/WFA combined treatment in oral cancer therapy.

## References

[1] Huang S.H., O’Sullivan B. (2013). Oral cancer: Current role of radiotherapy and chemotherapy. Med. Oral Patol. Oral Cir. Bucal.

[2] Moeller B.J., Richardson R.A., Dewhirst M.W. (2007). Hypoxia and radiotherapy: Opportunities for improved outcomes in cancer treatment. Cancer Metastasis Rev..

[3] Perry J.M., Tao F., Roy A., Lin T., He X.C., Chen S., Lu X., Nemechek J., Ruan L., Yu X. (2020). Overcoming Wnt-beta-catenin dependent anticancer therapy resistance in leukaemia stem cells. Nat. Cell Biol..

[4] Saga R., Matsuya Y., Takahashi R., Hasegawa K., Date H., Hosokawa Y. (2019). Analysis of the high-dose-range radioresistance of prostate cancer cells, including cancer stem cells, based on a stochastic model. J. Radiat. Res..

[5] Lee T.W., Wong W.W., Dickson B.D., Lipert B., Cheng G.J., Hunter F.W., Hay M.P., Wilson W.R. (2019). Radiosensitization of head and neck squamous cell carcinoma lines by DNA-PK inhibitors is more effective than PARP-1 inhibition and is enhanced by SLFN11 and hypoxia. Int. J. Radiat. Biol..

[6] Affolter A., Samosny G., Heimes A.S., Schneider J., Weichert W., Stenzinger A., Sommer K., Jensen A., Mayer A., Brenner W. (2017). Multikinase inhibitors sorafenib and sunitinib as radiosensitizers in head and neck cancer cell lines. Head Neck.

[7] Omidi B.R., Gosili A., Jaber-Ansari M., Mahdkhah A. (2018). Intensity output and effectiveness of light curing units in dental offices. J. Clin. Exp. Dent..

[8] Coohill T.P., Sagripanti J.L. (2009). Bacterial inactivation by solar ultraviolet radiation compared with sensitivity to 254 nm radiation. Photochem. PhotoBiol..

[9] Yamauchi T., Adachi S., Yasuda I., Nakashima M., Kawaguchi J., Yoshioka T., Hirose Y., Kozawa O., Moriwaki H. (2011). Ultra-violet irradiation induces apoptosis via mitochondrial pathway in pancreatic cancer cells. Int. J. Oncol..

[10] Adachi S., Yasuda I., Nakashima M., Yamauchi T., Kawaguchi J., Shimizu M., Itani M., Nakamura M., Nishii Y., Yoshioka T. (2011). Ultraviolet irradiation can induce evasion of colon cancer cells from stimulation of epidermal growth factor. J. Biol. Chem..

[11] Bhattacharya S.K., Satyan K.S., Ghosal S. (1997). Antioxidant activity of glycowithanolides from *Withania somnifera*. Indian J. Exp. Biol..

[12] Devkar S.T., Jagtap S.D., Katyare S.S., Hegde M.V. (2014). Estimation of antioxidant potential of individual components present in complex mixture of *Withania somnifera* (Ashwagandha) root fraction by thin-layer chromatography-2,2-diphenyl-1-picrylhdrazyl method. J. Planar Chromatogr..

[13] Yang I.-H., Kim L.-H., Shin J.-A., Cho S.-D. (2015). Chemotherapeutic effect of withaferin A in human oral cancer cells. J. Cancer Ther..

[14] Chang H.W., Li R.N., Wang H.R., Liu J.R., Tang J.Y., Huang H.W., Chan Y.H., Yen C.Y. (2017). Withaferin A induces oxidative stress-mediated apoptosis and DNA damage in oral cancer cells. Front. Physiol..

[15] Munagala R., Kausar H., Munjal C., Gupta R.C. (2011). Withaferin A induces p53-dependent apoptosis by repression of HPV oncogenes and upregulation of tumor suppressor proteins in human cervical cancer cells. Carcinogenesis.

[16] Li X., Zhu F., Jiang J., Sun C., Wang X., Shen M., Tian R., Shi C., Xu M., Peng F. (2015). Synergistic antitumor activity of withaferin A combined with oxaliplatin triggers reactive oxygen species-mediated inactivation of the PI3K/AKT pathway in human pancreatic cancer cells. Cancer Lett..

[17] Hsu J.H., Chang P.M., Cheng T.S., Kuo Y.L., Wu A.T., Tran T.H., Yang Y.H., Chen J.M., Tsai Y.C., Chu Y.S. (2019). Identification of withaferin A as a potential candidate for anti-cancer therapy in non-small cell lung cancer. Cancers.

[18] Xia S., Miao Y., Liu S. (2018). Withaferin A induces apoptosis by ROS-dependent mitochondrial dysfunction in human colorectal cancer cells. Biochem. Biophys. Res. Commun..

[19] Sari A.N., Bhargava P., Dhanjal J.K., Putri J.F., Radhakrishnan N., Shefrin S., Ishida Y., Terao K., Sundar D., Kaul S.C. (2020). Combination of withaferin-A and CAPE provides superior anticancer potency: Bioinformatics and experimental evidence to their molecular targets and mechanism of action. Cancers.

[20] Tsugeno Y., Sato F., Muragaki Y., Kato Y. (2014). Cell culture of human gingival fibroblasts, oral cancer cells and mesothelioma cells with serum-free media, STK1 and STK2. Biomed. Rep..

[21] Wang H.R., Tang J.Y., Wang Y.Y., Farooqi A.A., Yen C.Y., Yuan S.F., Huang H.W., Chang H.W. (2019). Manoalide preferentially provides antiproliferation of oral cancer cells by oxidative stress-mediated apoptosis and DNA damage. Cancers.

[22] Yeh C.C., Tseng C.N., Yang J.I., Huang H.W., Fang Y., Tang J.Y., Chang F.R., Chang H.W. (2012). Antiproliferation and induction of apoptosis in Ca9-22 oral cancer cells by ethanolic extract of *Gracilaria tenuistipitata*. Molecules.

[23] Chen C.Y., Yen C.Y., Wang H.R., Yang H.P., Tang J.Y., Huang H.W., Hsu S.H., Chang H.W. (2016). Tenuifolide B from *Cinnamomum tenuifolium* stem selectively inhibits proliferation of oral cancer cells via apoptosis, ROS generation, mitochondrial depolarization, and DNA damage. Toxins.

[24] Liu P.F., Tsai K.L., Hsu C.J., Tsai W.L., Cheng J.S., Chang H.W., Shiau C.W., Goan Y.G., Tseng H.H., Wu C.H. (2018). Drug repurposing screening identifies tioconazole as an ATG4 inhibitor that suppresses autophagy and sensitizes cancer cells to chemotherapy. Theranostics.

[25] Chang H.W., Tang J.Y., Yen C.Y., Chang H.S., Huang H.W., Chung Y.A., Chen I.S., Huang M.Y. (2016). Synergistic anti-oral cancer effects of UVC and methanolic extracts of *Cryptocarya concinna* roots via apoptosis, oxidative stress and DNA damage. Int. J. Radiat. Biol..

[26] Vignon C., Debeissat C., Georget M.T., Bouscary D., Gyan E., Rosset P., Herault O. (2013). Flow cytometric quantification of all phases of the cell cycle and apoptosis in a two-color fluorescence plot. PLoS ONE.

[27] Tang J.Y., Xu Y.H., Lin L.C., Ou-Yang F., Wu K.H., Tsao L.Y., Yu T.J., Huang H.W., Wang H.R., Liu W. (2019). LY303511 displays antiproliferation potential against oral cancer cells in vitro and in vivo. Environ. Toxicol..

[28] Wang S.C., Wang Y.Y., Lin L.C., Chang M.Y., Yuan S.F., Tang J.Y., Chang H.W. (2020). Combined treatment of sulfonyl chromen-4-ones (CHW09) and ultraviolet-C (UVC) enhances proliferation inhibition, apoptosis, oxidative stress, and DNA damage against oral cancer cells. Int. J. Mol. Sci..

[29] Chang Y.T., Huang C.Y., Tang J.Y., Liaw C.C., Li R.N., Liu J.R., Sheu J.H., Chang H.W. (2017). Reactive oxygen species mediate soft corals-derived sinuleptolide-induced antiproliferation and DNA damage in oral cancer cells. OncoTargets Ther..

[30] Huang H.W., Tang J.Y., Ou-Yang F., Wang H.R., Guan P.Y., Huang C.Y., Chen C.Y., Hou M.F., Sheu J.H., Chang H.W. (2018). Sinularin selectively kills breast cancer cells showing G2/M arrest, apoptosis, and oxidative DNA damage. Molecules.

[31] Tang J.Y., Wu C.Y., Shu C.W., Wang S.C., Chang M.Y., Chang H.W. (2018). A novel sulfonyl chromen-4-ones (CHW09) preferentially kills oral cancer cells showing apoptosis, oxidative stress, and DNA damage. Environ. Toxicol..

[32] Yen C.Y., Hou M.F., Yang Z.W., Tang J.Y., Li K.T., Huang H.W., Huang Y.H., Lee S.Y., Fu T.F., Hsieh C.Y. (2015). Concentration effects of grape seed extracts in anti-oral cancer cells involving differential apoptosis, oxidative stress, and DNA damage. BMC Complement. Altern. Med..

[33] Tang J.Y., Farooqi A.A., Ou-Yang F., Hou M.F., Huang H.W., Wang H.R., Li K.T., Fayyaz S., Shu C.W., Chang H.W. (2018). Oxidative stress-modulating drugs have preferential anticancer effects—Involving the regulation of apoptosis, DNA damage, endoplasmic reticulum stress, autophagy, metabolism, and migration. Semin. Cancer Biol..

[34] Huang C., Li J., Ding M., Leonard S.S., Wang L., Castranova V., Vallyathan V., Shi X. (2001). UV Induces phosphorylation of protein kinase B (Akt) at Ser-473 and Thr-308 in mouse epidermal Cl 41 cells through hydrogen peroxide. J. Biol. Chem..

[35] Peng S.Y., Lin L.C., Yang Z.W., Chang F.R., Cheng Y.B., Tang J.Y., Chang H.W. (2020). Combined treatment with low cytotoxic ethyl acetate Nepenthes extract and ultraviolet-C improves antiproliferation to oral cancer cells via oxidative stress. Antioxidants.

[36] Sharada A.C., Solomon F.E., Devi P.U., Udupa N., Srinivasan K.K. (1996). Antitumor and radiosensitizing effects of withaferin A on mouse Ehrlich ascites carcinoma in vivo. Acta Oncol..

[37] Devi P.U., Kamath R., Rao B.S. (2000). Radiosensitization of a mouse melanoma by withaferin A: In vivo studies. Indian J. Exp. Biol..

[38] Yang E.S., Choi M.J., Kim J.H., Choi K.S., Kwon T.K. (2011). Combination of withaferin A and X-ray irradiation enhances apoptosis in U937 cells. Toxicol. Vitr..

[39] Kawaguchi J., Adachi S., Yasuda I., Yamauchi T., Nakashima M., Ohno T., Shimizu M., Yoshioka T., Itani M., Kozawa O. (2012). Cisplatin and ultra-violet-C synergistically down-regulate receptor tyrosine kinases in human colorectal cancer cells. Mol. Cancer.

[40] Robin T., Capes-Davis A., Bairoch A. (2020). CLASTR: The Cellosaurus STR similarity search tool—A precious help for cell line authentication. Int. J. Cancer.

[41] Tan B.L., Norhaizan M.E., Liew W.P., Sulaiman Rahman H. (2018). Antioxidant and oxidative stress: A mutual interplay in age-related diseases. Front. Pharmacol..

[42] Mathur S., Kaur P., Sharma M., Katyal A., Singh B., Tiwari M., Chandra R. (2004). The treatment of skin carcinoma, induced by UV B radiation, using 1-oxo-5beta, 6beta-epoxy-witha-2-enolide, isolated from the roots of *Withania somnifera*, in a rat model. Phytomedicine.

[43] Braun L., Cohen M. (2015). Herbs and Natural Supplements, Volume 2: An. Evidence-Based Guide.

[44] Malik F., Kumar A., Bhushan S., Khan S., Bhatia A., Suri K.A., Qazi G.N., Singh J. (2007). Reactive oxygen species generation and mitochondrial dysfunction in the apoptotic cell death of human myeloid leukemia HL-60 cells by a dietary compound withaferin A with concomitant protection by N-acetyl cysteine. Apoptosis.

[45] Bouayed J., Bohn T. (2010). Exogenous antioxidants--Double-edged swords in cellular redox state: Health beneficial effects at physiologic doses versus deleterious effects at high doses. Oxid. Med. Cell Longev..

[46] Chan W.H., Yu J.S. (2000). Inhibition of UV irradiation-induced oxidative stress and apoptotic biochemical changes in human epidermal carcinoma A431 cells by genistein. J. Cell Biochem..

[47] Yu T.J., Tang J.Y., Ou-Yang F., Wang Y.Y., Yuan S.F., Tseng K., Lin L.C., Chang H.W. (2020). Low concentration of withaferin A inhibits oxidative stress-mediated migration and invasion in oral cancer cells. Biomolecules.

[48] Cadet J., Sage E., Douki T. (2005). Ultraviolet radiation-mediated damage to cellular DNA. Mutat. Res..

[49] Evans M.D., Cooke M.S., Podmore I.D., Zheng Q., Herbert K.E., Lunec J. (1999). Discrepancies in the measurement of UVC-induced 8-oxo-2′-deoxyguanosine: Implications for the analysis of oxidative DNA damage. Biochem. Biophys. Res. Commun..

[50] Turinetto V., Giachino C. (2015). Multiple facets of histone variant H2AX: A DNA double-strand-break marker with several biological functions. Nucleic Acids Res..

[51] Dunkern T.R., Fritz G., Kaina B. (2001). Ultraviolet light-induced DNA damage triggers apoptosis in nucleotide excision repair-deficient cells via Bcl-2 decline and caspase-3/-8 activation. Oncogene.

[52] Ly J.D., Grubb D.R., Lawen A. (2003). The mitochondrial membrane potential (deltapsi(m)) in apoptosis; an update. Apoptosis.

[53] Bairoch A. (2018). The Cellosaurus, a Cell-Line Knowledge Resource. J. Biomol. Tech..

